# Da-Cheng-Qi Decoction Alleviates Intestinal Injury in Rats with Severe Acute Pancreatitis by Inhibiting the JAK2-STAT3 Signaling Pathway

**DOI:** 10.1155/2019/3909468

**Published:** 2019-08-14

**Authors:** Wenyin Jin, Yinfeng Shen

**Affiliations:** Department of Surgery, Hubei Hospital of Chinese Medicine, Hubei University of Chinese Medicine, Wuhan, China

## Abstract

**Objective:**

To investigate the effect of Da-Cheng-Qi decoction (DCQD) on treating intestinal injury in rats with severe acute pancreatitis (SAP), based on the Janus kinase 2 (JAK2)/signal transducers and transcription 3 (STAT3) signaling pathway.

**Methods:**

Rats were randomly divided into the SAP group, SAP + ruxolitinib (JAK2 inhibitor) group, SAP + Stattic (STAT3 inhibitor) group, SAP + DCQD group, and sham operation group. They were further divided into 3-hour, 6-hour, 12-hour, and 18-hour subgroups. Levels of amylase and the inflammatory cytokines tumor necrosis factor-*α*, interleukin 6, interleukin 10, and interleukin 4 in plasma were tested. The messenger ribonucleic acid (mRNA) expression of JAK2 and STAT3 and the protein expression of phosphorylated JAK2 (p-JAK2) and phosphorylated STAT3 (p-STAT3) in the pancreas and terminal ileum tissues were examined.

**Results:**

Rats with SAP had severe changes in plasma levels of amylase and inflammatory cytokines and showed an overexpression of JAK2 mRNA, STAT3 mRNA, p-JAK2 protein, and p-STAT3 protein in the pancreas and terminal ileum. The events could be downregulated by treatment with DCQD, JAK2 inhibitor, and STAT3 inhibitor.

**Conclusions:**

In rats with SAP, DCQD ameliorated inflammatory cytokines and intestinal injury, which may be closely associated with the inhibition of the JAK2/STAT3 signaling pathway.

## 1. Introduction

Acute pancreatitis is an inflammatory disease of the pancreas caused by the production and release of various inflammatory cytokines. Approximately 20% of patients with acute pancreatitis develop severe acute pancreatitis (SAP) [1], which is a common and potentially fatal disease and is characterized by a mortality risk of 10%–30% and various complications [2]. In the early stage of SAP, the cause of mortality is closely associated with single or multiple organ complications such as lung injury and intestine barrier functional disturbance [3]. In particular, intestinal injury is one of the most frequent complications of this severe disease, and extensive research demonstrates that it contributes significantly to high morbidity and mortality [4, 5].

Janus kinase/signal transducers and transcription (JAK/STAT) signaling constitutes a major pathway for cytokine signal transduction, which is involved in inflammation and in the beneficial and protective role of JAK2 and STAT3 in inflammatory responses [6, 7]. Tumor necrosis factor-*α* (TNF-*α*) and interleukin 6 (IL-6) are important proinflammatory cytokines. They can stimulate the inflammatory cascade and amplify inflammatory effects, which is a main reason for organ injury and multiple organ dysfunction syndrome during SAP [8]. By contrast, the levels of anti-inflammatory factors such as interleukin 10 (IL-10) and interleukin 4 (IL-4) are also increased, which compensate for the inflammatory response [8].

For decades, Da-Cheng-Qi decoction (DCQD) has been widely used to treat SAP effectively in clinical settings in China. DCQD, a special type of treatment for selective gut decontamination [9], is composed of *Rheum palmatum L*. (Dahuang), *Magnolia henryi Dunn*. (Houpu), *Citrus aurantium L*. (Zhishi), and Natrii Sulphas (Mangxiao).

However, the molecular mechanisms of the systemic inflammatory response on the JAK2/STAT3 signaling pathway in SAP are not well understood. The precise mechanisms of JAK2/STAT3 pathway inhibitors in SAP in relation to pancreatic diseases and intestinal injury remain largely unknown. To determine whether DCQD can alleviate the systemic inflammatory response and intestinal injury associated with SAP via regulating the JAK2-STAT3 signaling pathway, we established a controlled SAP survival rat model; used DCQD, ruxolitinib as a JAK2 inhibitor and Stattic, a STAT3 inhibitor, as an intervention; detected the dynamic levels of the inflammatory cytokines TNF-*α*, IL-6, IL-4, and IL-10 in plasma; and evaluated the gene expression of JAK2 and STAT3 in the pancreas and terminal ileum.

## 2. Materials and Methods

### 2.1. Animals

Healthy male Wistar rats (weighing 200–250 g) were purchased from the Experimental Animal Center of Hubei Province (Wuhan, China). Animal experimental protocols were conducted in accordance with the regulations for the Administration of Affairs Concerning Experimental Animals of Hubei Province. All rats were housed in standard room temperature (22°C) under a 12-hour light/dark cycle. They had free access to standard rat chow and tap drinking water before the experiments.

### 2.2. Anesthetics and Taurocholate-Induced Pancreatitis

The animals were fasted overnight before the experiment, but they were allowed free access to drinking water. Rats were anesthetized with an intraperitoneal injection of 3% pentobarbital sodium (40 mg/kg body weight; Sigma-Aldrich China, Inc., Shanghai, China). Surgical procedures were conducted under aseptic conditions. The rat model of SAP was prepared using the method of Aho et al. [10]. After entering the abdomen, anatomical structures were identified. An intravenous catheter (Suzhou Linhwa Medical Devices Co., Ltd., Suzhou, China) was inserted into the biliopancreatic duct through the duodenum. A soft microvascular clamp was used to close the liver hilum to prevent the infused material from entering the liver. Sodium taurocholate (5%; 1.0 mL/kg body weight, 0.1 mL/min; Sigma-Aldrich China, Inc., Shanghai, China) was injected into the bile-pancreatic duct. Five minutes later, the microvascular clamp and epidural catheter were removed, and the median epigastric incision was closed.

### 2.3. Preparation of DCQD

The Da-Cheng-Qi decoction formula comes from the classic Chinese medicine book *Shang Han Lun*, which describes the dosages of the components as follows: 12 g of Dahuang, 24 g of Houpu, 12 g of Zhishi, and 9 g of Mangxiao. The DCQD spray-dried drug powders were purchased from Hubei Tianji Traditional Chinese Medicine Pieces Co., Ltd. (Hubei, China). The crude formula components were extracted, concentrated, and used as described by Zhang et al. [11]. The spray-dried powders (i.e., Dahuang, Houpu, Zhishi, and Mangxiao) were in the standard ratio of 12 : 24 : 12 : 9 and were mixed and reconstituted with sterile distilled water at 1.2 g/mL of DCQD concentration for the crude drug.

### 2.4. Experimental Design

In the pre-experiments, the rats were euthanized at 0 hour, 3 hours, 6 hours, 12 hours, 18 hours, and 24 hours after the operation (*n* = 12 per group). The zero timepoint is the point of the first injection of sodium taurocholate. The first series of experiments was 24 hours. In the experiments, a successful pancreatitis model was confirmed. We selected 3 hours, 6 hours, 12 hours, and 18 hours as the designated timepoints in the following experiments by evaluating the trends of these measurements.

Rats were randomly divided into the SAP group, SAP + ruxolitinib group (R group), SAP + Stattic group (S group), SAP + DCQD group (DCQD group), and sham operation group (SO group). Ruxolitinib and Stattic were purchased from Selleck Chemicals, Houston, TX, USA. The groups were also randomly divided into 3-hour, 6-hour, 12-hour, and 18-hour subgroups (*n* = 12 per subgroup). In the SO group (*n* = 48), the same amount of saline instead of sodium taurocholate was injected into the bile-pancreatic duct, and the other procedures were the same as for the SAP rats. The rats in the R group were intragastrically administered 180 mg/kg ruxolitinib within 2 hours before the operation, whereas the rats in the other groups were intragastrically administered an equal amount of 0.9% sodium chloride. The rats in the S group were injected intraperitoneally with Stattic (3.75 mg/kg) within 2 hours before the operation, whereas the rats in the other groups were injected with an equal amount of saline. The rats in the DCQD group were intragastrically administered 12 g/kg DCQD within 2 hours before the operation, whereas the rats in the other groups were intragastrically administered an equal amount of saline.

### 2.5. Collection of Specimens

After re-anesthesia for the surviving rats, the test subjects were operated on at different timepoints, and tissue samples (e.g., abdominal aorta blood, pancreas, and terminal ileum) were obtained. Blood samples were maintained at 4°C for 10 minutes and centrifuged at 4°C at 3000 g for 10 minutes. The sera were stored at −80°C. Pancreas and terminal ileum tissue were washed in 0.9% sodium chloride at 4°C and stored at −80°C.

### 2.6. Serum Amylase Activity and Inflammatory Cytokine Assay

An Amy kit was used in an automated clinical biochemistry analysis unit (Hitachi Co., Tokyo, Japan) to evaluate serum amylase activity. The levels of TNF-*α*, IL-6, IL-4, and IL-10 in the serum samples were tested by an enzyme-linked immunosorbent assay, based on procedures described in the manufacturer's instruction manual (Wuhan Boster Biological Technology, Ltd., Wuhan, China).

### 2.7. Reverse Transcription-Quantitative Polymerase Chain Reaction

Twenty-four hours after surgery, the total cellular ribonucleic acid (RNA) was extracted from the tissue cells using the RNAiso Plus extraction reagent (Wuhan Boster Biological Technology, Ltd.), based on the manufacturer's protocol. Reverse transcription-quantitative polymerase chain reaction (RT-qPCR) was used to quantify the messenger RNA (mRNA) levels of JAK2 and STAT3 by using the Maxima SYBR-Green/ROX qPCR Master Mix (2×; Thermo Fisher Scientific, Inc., Waltham, MA, USA). The RNA was quantified by measuring the absorbance at 260 nm (Ultrospec 2100 Pro spectrophotometer; GE Healthcare, Buckinghamshire, UK). The concentration was 220–280 ng/*μ*L (based on the optical density (OD) of 260/280). The iScript cDNA synthesis kit (Bio-Rad Laboratories, Inc., Hercules, CA, USA) was used to synthesize complementary deoxyribonucleic acid (cDNA) from 1 *μ*L of RNA. The cDNA (500 ng) was amplified and detected using the Rotor-Gene 3000 sequence detection system (Qiagen, Inc., Valencia, CA, USA).

The primers and probes (Wuhan Boster Biological Technology, Ltd.) were as follows: JAK2 forward, 5′-TTT GAA GAC AGG GAC CCT ACA CAG -3′; JAK2 reverse, 5′-TCA TAG CGG CAC ATC TCC ACA-3′; STAT3 forward, 5′-CAC CCA TAG TGA GCC CTT GGA-3′; STAT3 reverse, 5′-TGA GTG CAG TGA CCA GGA CAG A-3′; glyceraldehyde-3-phosphate dehydrogenase (GAPDH) forward, 5′-CAA GGT CAT CCA TGA CAA CTT TG-3′; and GAPDH reverse, 5′-GTC CAC CAC CCT GTT GCT GTA G-3′. The internal control was GAPDH. Gene expression was calculated by using the 2-ΔΔCt method. The copy ratios of JAK2/GAPDH and STAT3/GAPDH were calculated as the relative expression levels. The reaction condition of PCR was as follows: denaturation at 95°C for 3 minutes, followed by 40 cycles of denaturation (95°C, 15 seconds), annealing (60°C, 30 seconds), and extension (72°C, 30 seconds).

### 2.8. Western Blotting Analysis

Ice-cold lysis buffer was used to collect total protein from frozen pancreas and terminal ileum, respectively. The protein (10 *μ*g) lysate and buffer were mixed and subjected to sodium dodecyl sulfate-polyacrylamide gel electrophoresis and later transferred to a nitrocellulose membrane (Wuhan Boster Biological Technology, Ltd.). Protein was blocked for 1 hour at 22°C with phosphate buffered saline (PBS) containing 5% skim milk powder and then immersed with primary antibodies (Wuhan Boster Biological Technology, Ltd.) at 4°C overnight. The membrane was incubated with horseradish peroxidase-conjugated secondary antibody (Wuhan Boster Biological Technology, Ltd.) for 1 hour at 20°C after three rinses with PBS. After rinsing 3 times with PBS, the membrane was developed with the electrochemiluminescence solution and exposed to the image using a gel imaging system (Bio-Rad, CA, USA). The OD of each band was detected using the Gel-Pro Analyzer 4.0 software (Media Cybernetics, Inc., Rockville, MD, USA).

Phosphorylated JAK2 (p-JAK2) and phosphorylated STAT3 (p-STAT3) levels were measured with GAPDH as a loading control. The relative target protein expression was the ratio of the target/the GAPDH band.

### 2.9. Statistical Analysis

The software package SPSS 11 (SPSS, Inc., Chicago, IL, USA) was used to analyze the data. Continuous data are expressed as the mean ± standard deviation. The nonparametric Mann–Whitney *U* test and the *χ*^2^ test with Yates correction were used to compare the continuous variables and the categorical variables, respectively, between the two groups. Comparisons of multiple groups were analyzed with one-way analysis of variance. *A* value of *P* < 0.05 was statistically significant.

## 3. Results

### 3.1. Serum Amylase

The levels of serum amylase were significantly increased at 3 hours in the SO and SAP groups and at 12 hours in the R, S, and DCQD groups (*P* < 0.05; Table 1). In the SAP group, amylase levels slowly decreased after peaking at 6 hours (*P* < 0.05; Table 1). In the R, S, and DCQD groups, the amylase levels peaked at 12 hours and thereafter slowly decreased (*P* < 0.05, Table 1). The pairwise comparisons between the SAP, R, S, and DCQD groups at four timepoints revealed that the amylase levels were less in the DCQD group than in the R group, less in the R group than in the S group, and less in the S group than in the SAP group (*P* < 0.05; Table 1).

### 3.2. Inflammatory Factors

The serum levels of the inflammatory cytokines TNF-*α*, IL-6, IL-10, and IL-4 peaked at 12 hours in the five groups (*P* < 0.05, Tables 2–5). After the peak, the proinflammatory and anti-inflammatory cytokines levels slowly decreased by 18 hours in the DCQD, R, and S groups (*P* < 0.05; Tables 2–5). In the SAP, R, S, and DCQD groups, the cytokine levels were increased at 6 hours and 12 hours (*P* < 0.05; Tables 2–5) and rapidly decreased by 18 hours, especially in the SAP group (*P* < 0.05; Tables 2–5). The pairwise comparisons between the three SAP groups at all experimental timepoints revealed that the cytokine levels were greater in the SAP group than in the S group, greater in the S group than in the R group, and greater in the R group than in the DCQD group (*P* < 0.05; Tables 2–5).

### 3.3. JAK2 and STAT3 mRNA Expression Levels

**Figure 1:**
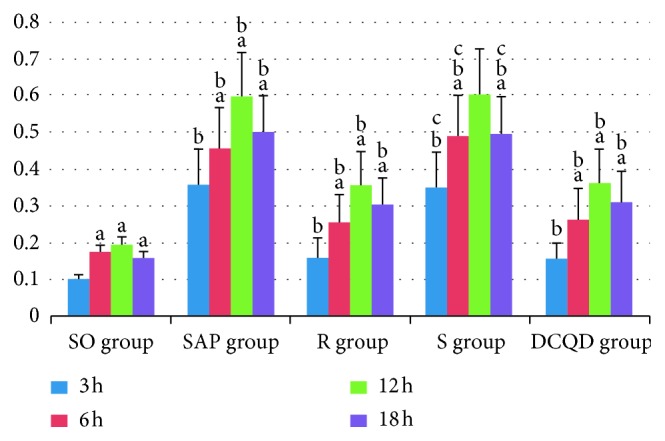
The expression of JAK2 mRNA in the pancreas. (a) In the SO, SAP, R, S, and DCQD groups, differences between 3 hours versus 6 hours, 6 hours versus 12 hours, and 12 hours versus 18 hours are significant (*P* < 0.05). (b) At 3 hours, 6 hours, 12 hours, and 18 hours, the SO group versus the SAP group, the SAP group versus the R group, the R group versus the S group, and the S group versus the DCQD group are significantly different (*P* < 0.05). (c) At the same timepoints, differences between the SAP group versus the S group are not significant (*P* > 0.05). DCQD, Da-Cheng-Qi decoction; GAPDH, glyceraldehyde-3-phosphate dehydrogenase; JAK2, Janus kinase 2; mRNA, messenger ribonucleic acid; p-JAK2, phosphorylated Janus kinase 2; p-STAT3, phosphorylated signal transducers and transcription 3; R, ruxolitinib-treated rats with severe acute pancreatitis; S, Stattic-treated rats with severe acute pancreatitis; SAP, severe acute pancreatitis; SO, sham operation; STAT3, signal transducers and transcription 3.

**Figure 2:**
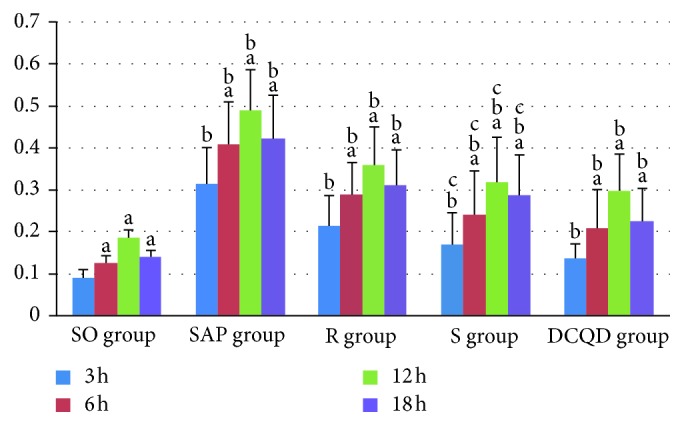
The expression of JAK2 mRNA in the terminal ileum. (a) In the SO, SAP, R, S, and DCQD groups, differences between 3 hours versus 6 hours, 6 hours versus 12 hours, and 12 hours versus 18 hours are significant (*P* < 0.05). (b) At 3 hours, 6 hours, 12 hours, and 18 hours, differences between the SO group versus the SAP group, the SAP group versus the R group, the R group versus the S group, and the S group versus the DCQD group are significant (*P* < 0.05). (c) At the same timepoints, the SAP group versus the S group is not significantly different (*P* > 0.05). DCQD, Da-Cheng-Qi decoction; GAPDH, glyceraldehyde-3-phosphate dehydrogenase; JAK2, Janus kinase 2; mRNA, messenger ribonucleic acid; p-JAK2, phosphorylated Janus kinase 2; p-STAT3, phosphorylated signal transducers and transcription 3; R, ruxolitinib-treated rats with severe acute pancreatitis; S, Stattic-treated rats with severe acute pancreatitis; SAP, severe acute pancreatitis; SO, sham operation; STAT3, signal transducers and transcription 3.

The levels of JAK2 mRNA in the pancreas and terminal ileum were increased significantly at 3 hours and 6 hours and peaked at 12 hours in the five groups (*P* < 0.05; Figures 1 and 2). For the SAP group versus the S group and for the R group versus the DCQD group, the dynamic JAK2 mRNA expression levels in the pancreas and terminal ileum changed consistently at the same timepoints (*P* > 0.05; Figures 1 and 2). The JAK2 mRNA expression levels were markedly elevated in the pancreas and terminal ileum in the SAP and S groups and peaked at 12 hours (*P* < 0.05; Figures 1 and 2). After peaking at 12 hours, the JAK2 mRNA expression levels in the pancreas and terminal ileum slowly decreased in the five groups (*P* < 0.05; Figures 1 and 2). The pairwise comparisons among the SAP, R, S, and DCQD groups at the four timepoints revealed that the JAK2 mRNA expression in the pancreas and terminal ileum was less in the R group than in the SAP group (*P* < 0.05; Figures 1 and 2).

**Figure 3:**
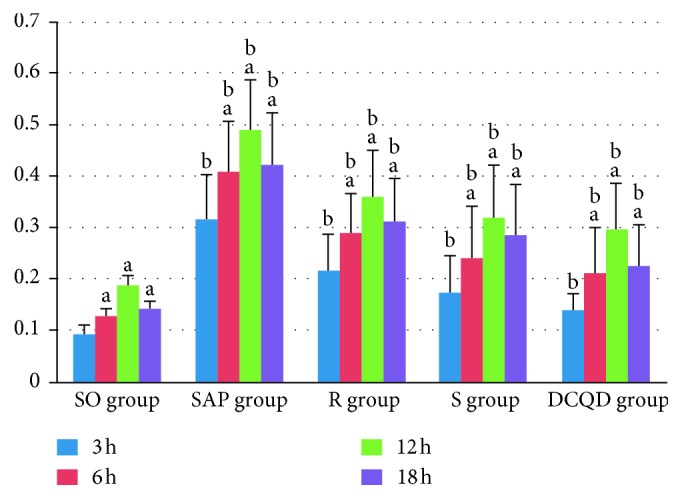
The expression of STAT3 mRNA in the pancreas. (a) In the five groups, differences between 3 hours versus 6 hours, 6 hours versus 12 hours, and 12 hours versus 18 hours are significant (*P* < 0.05). (b) At four timepoints, differences between the SO group versus the SAP group, the SAP group versus the R group, the R group versus the S group, and the S group versus the DCQD group are significant (*P* < 0.05). DCQD, Da-Cheng-Qi decoction; GAPDH, glyceraldehyde-3-phosphate dehydrogenase; JAK2, Janus kinase 2; mRNA, messenger ribonucleic acid; p-JAK2, phosphorylated Janus kinase 2; p-STAT3, phosphorylated signal transducers and transcription 3; R, ruxolitinib-treated rats with severe acute pancreatitis; S, Stattic-treated rats with severe acute pancreatitis; SAP, severe acute pancreatitis; SO, sham operation; STAT3, signal transducers and transcription 3.

**Figure 4:**
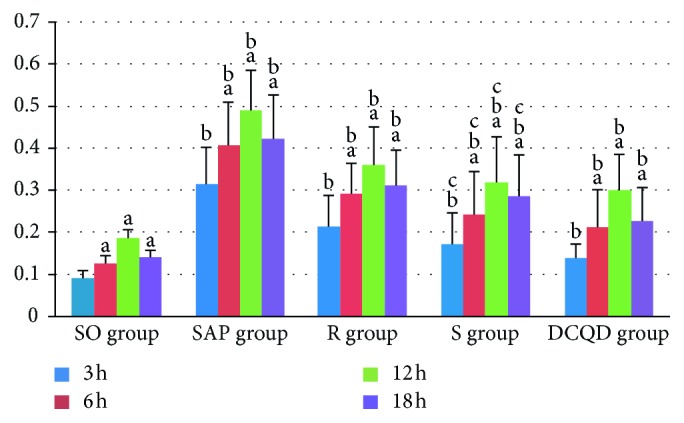
The expression of STAT3 mRNA in the terminal ileum. (a) In the five groups, differences between 3 hours versus 6 hours, 6 hours versus 12 hours, and 12 hours versus 18 hours are significant (*P* < 0.05). (b) At 3 hours, 6 hours, 12 hours, and 18 hours, differences between the SO group versus the SAP group, the SAP group versus the R group, the R group versus the S group, and the S group versus the DCQD group are significant (*P* < 0.05). DCQD, Da-Cheng-Qi decoction; GAPDH, glyceraldehyde-3-phosphate dehydrogenase; JAK2, Janus kinase 2; mRNA, messenger ribonucleic acid; p-JAK2, phosphorylated Janus kinase 2; p-STAT3, phosphorylated signal transducers and transcription 3; R, ruxolitinib-treated rats with severe acute pancreatitis; S, Stattic-treated rats with severe acute pancreatitis; SAP, severe acute pancreatitis; SO, sham operation; STAT3, signal transducers and transcription 3.

The STAT3 mRNA expression in the pancreas and terminal ileum peaked at 12 hours in the five groups (*P* < 0.05; Figures 3 and 4). After the peak, the STAT3 mRNA expression slowly declined by 18 hours in the five groups (*P* < 0.05; Figures 3 and 4). In the SAP, R, S, and DCQD groups, the STAT3 mRNA expression level increased quickly at 6 hours and 12 hours (*P* < 0.05; Figures 3 and 4) and rapidly at 18 hours, especially in the SAP group (*P* < 0.05; Figures 3 and 4). The pairwise comparisons between all SAP groups at all timepoints revealed the levels of STAT3 mRNA expression in the pancreas and terminal ileum were greater in the SAP group than in the S group, greater in the S group than in the R group, and greater in the R group than in the DCQD group (*P* < 0.05; Figures 3 and 4).

### 3.4. Protein Expression of p-JAK2 and p-STAT3

**Figure 5:**
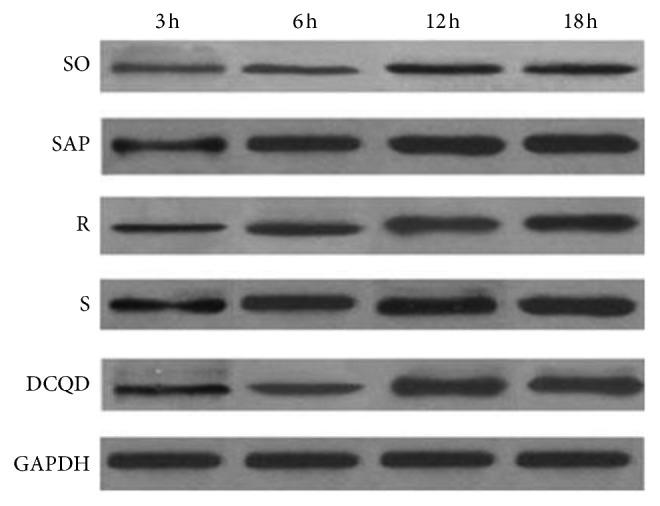
The p-JAK2 protein in the pancreas. DCQD, Da-Cheng-Qi decoction; GAPDH, glyceraldehyde-3-phosphate dehydrogenase; JAK2, Janus kinase 2; mRNA, messenger ribonucleic acid; p-JAK2, phosphorylated Janus kinase 2; p-STAT3, phosphorylated signal transducers and transcription 3; R, ruxolitinib-treated rats with severe acute pancreatitis; S, Stattic-treated rats with severe acute pancreatitis; SAP, severe acute pancreatitis; SO, sham operation; STAT3, signal transducers and transcription 3.

**Figure 6:**
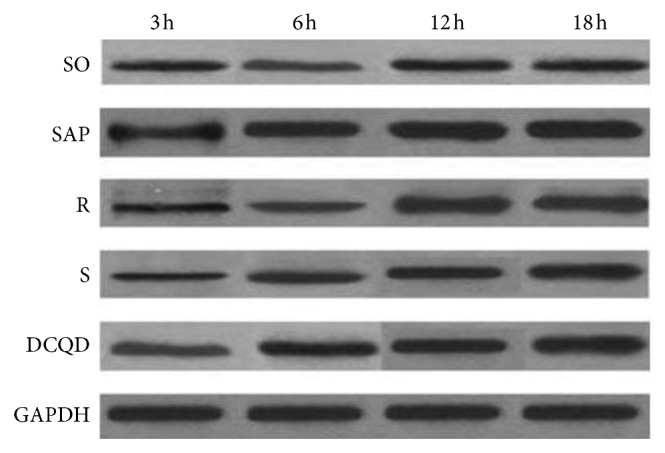
The p-JAK2 protein in the terminal ileum. DCQD, Da-Cheng-Qi decoction; GAPDH, glyceraldehyde-3-phosphate dehydrogenase; JAK2, Janus kinase 2; mRNA, messenger ribonucleic acid; p-JAK2, phosphorylated Janus kinase 2; p-STAT3, phosphorylated signal transducers and transcription 3; R, ruxolitinib-treated rats with severe acute pancreatitis; S, Stattic-treated rats with severe acute pancreatitis; SAP, severe acute pancreatitis; SO, sham operation; STAT3, signal transducers and transcription 3.

The levels of p-JAK2 protein in the pancreas and terminal ileum were elevated significantly at 3 hours and 6 hours and peaked at 12 hours in the five groups (*P* < 0.05; Figures 5 and 6, Tables6 and 7). Comparisons of the SAP group versus the S group and the R group versus the DCQD group revealed that the dynamic p-JAK2 protein expression levels in the pancreas and terminal ileum were similar at the same timepoints (*P* > 0.05; Figures 5 and 6, Tables 6 and 7). The p-JAK2 protein expression levels in the pancreas and terminal ileum were elevated in the SAP and S groups and peaked at 12 hours (*P* < 0.05; Figures 5 and 6, Tables 6 and 7). In the five groups, after peaking at 12 hours, the p-JAK2 protein expression levels in the pancreas and terminal ileum declined slowly (*P* < 0.05; Figures 5 and 6, Tables 6 and 7). The pairwise comparisons between the SAP, R, S, and DCQD groups at the four timepoints revealed that the p-JAK2 protein expression levels in the pancreas and terminal ileum were varied with the R group less than the SAP group (*P* < 0.05; Figures 5 and 6, Tables 6 and 7).

**Figure 7:**
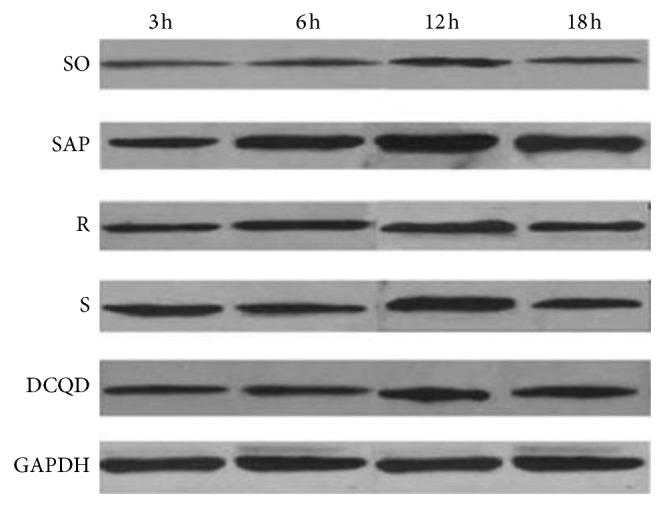
The p-STAT3 protein in the pancreas. DCQD, Da-Cheng-Qi decoction; GAPDH, glyceraldehyde-3-phosphate dehydrogenase; JAK2, Janus kinase 2; mRNA, messenger ribonucleic acid; p-JAK2, phosphorylated Janus kinase 2; p-STAT3, phosphorylated signal transducers and transcription 3; R, ruxolitinib-treated rats with severe acute pancreatitis; S, Stattic-treated rats with severe acute pancreatitis; SAP, severe acute pancreatitis; SO, sham operation; STAT3, signal transducers and transcription 3.

**Figure 8:**
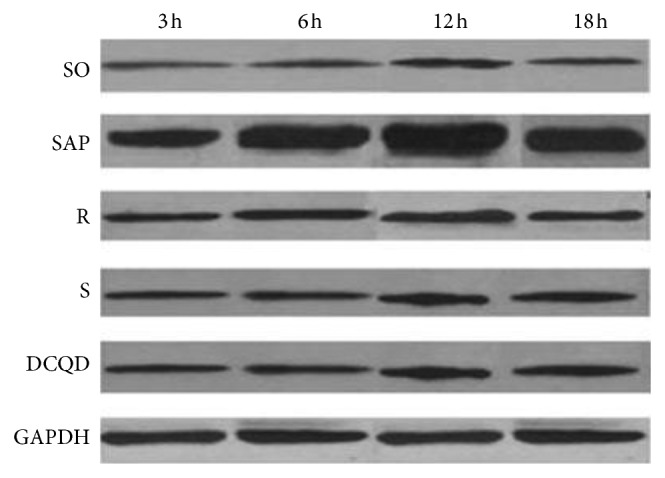
The p-STAT3 protein in the terminal ileum. DCQD, Da-Cheng-Qi decoction; GAPDH, glyceraldehyde-3-phosphate dehydrogenase; JAK2, Janus kinase 2; mRNA, messenger ribonucleic acid; p-JAK2, phosphorylated Janus kinase 2; p-STAT3, phosphorylated signal transducers and transcription 3; R, ruxolitinib-treated rats with severe acute pancreatitis; S, Stattic-treated rats with severe acute pancreatitis; SAP, severe acute pancreatitis; SO, sham operation; STAT3, signal transducers and transcription 3.

The levels of p-STAT3 protein in the pancreas and terminal ileum peaked at 12 hours in the five groups (*P* < 0.05; Figures 7 and 8, Tables 8 and 9). After peaking, the levels of p-STAT3 protein expression slowly declined in the five groups by 18 hours (*P* < 0.05; Figures 7 and 8, Tables 8 and 9). In the SAP, R, S, and DCQD groups, the p-STAT3 protein expression levels increased quickly at 6 hours and 12 hours (*P* < 0.05; Figures 7 and 8, Tables 8 and 9) and then decreased rapidly by 18 hours, especially in the SAP group (*P* < 0.05; Figures 7 and 8, Tables 8 and 9). The pairwise comparisons between the four SAP groups at the four timepoints revealed that the levels of p-STAT3 protein expression in the pancreas and terminal ileum were dynamic with the levels of the SAP group greater than those of the S group, the levels of the S group greater than those of the R group, and the levels of the R group greater than those of the DCQD group (*P* < 0.05; Figures 7 and 8, Tables 8 and 9).

## 4. Discussion

There were many research studies using the SAP model to study Chinese medicine for pancreatitis, which emphasized that Chinese Materia Medica could improve the gastrointestinal motility [12], reduce intestinal barrier dysfunction [13], inhibit intestinal bacteria and endotoxin translocation [13], regulate the inflammatory response [14], ameliorate the disease severity [15], alleviate the degree of organs injury [14, 15], and increase the activity and expression of Ca^2+^-Mg^2+^-ATPase in pancreatic acinar cells [16]. There were fewer studies for observation of the role of JAK2/STAT3 pathway in model rats with intestinal injury, and the role of DCQD in JAK2/STAT3 pathway.

In this study, we established a rat model of SAP to determine whether the DCQD could alleviate the systemic inflammatory response and intestinal injury associated with SAP via regulating JAK2-STAT3 signaling pathway. Our findings indicate that DCQD ameliorated inflammatory cytokines and intestinal injury in rats with SAP, which may be closely associated with the inhibition of the JAK2/STAT3 signaling pathway. SAP is an inflammatory disease with variable involvement of the pancreas and/or remote organ systems and with the development of a systemic inflammatory response [17]. The inflammatory mediators escalate the inflammatory response, which stimulates the inflammatory cascade and leads to systemic complications. Acute lung injury and/or intestinal injury are the most frequent and most serious systemic complications and may occur in all cases of SAP [18]. Severe complications are often intestinal barrier injury and lung injury. Intestinal barrier damage is normally associated with changes in mucosal autophagy and oxidative stress [19, 20] and permits great quantities of gut bacteria and endotoxins to enter the blood, and eventually to enter remote organs [21]. Increasing evidence has indicated that the pathological mechanisms of SAP and SAP-associated organ failure may occur because of proinflammatory cytokines [22].

Sodium taurocholate-induced pancreatitis in rats is the most well-characterized model of pancreatitis that has been extensively employed for probing the events of the evolution of pancreatitis [23]. The JAK-STAT pathway is a family of receptor-associated JAKs which phosphorylate tyrosine residues on STATs [24]. The JAK2-STAT3 pathway is the most important pathway in the JAK-STAT pathway family that is involved in the inflammatory immune response [25, 26].

Ruxolitinib is a tyrosine kinase inhibitor that is widely used to inhibit JAK1/2 and is approved by the US Food and Drug Administration (Silver Spring, MD, USA) for clinical use [27]. Stattic is a tool for inhibiting STAT3 in animal tumor models displaying constitutive STAT3 activation [28]. The main pharmacological components of DCQD are similar in the peripheral blood, pancreas, and intestine [11, 29].

Our findings suggested that rats with SAP had severe changes in the levels of amylase and inflammatory cytokines in the plasma and the overexpression of JAK2 mRNA, STAT3 mRNA, p-JAK2 protein, and p-STAT3 protein in the pancreas and terminal ileum. A JAK2 inhibitor and STAT3 inhibitor can obviously downregulate these events. The pairwise comparisons between the SAP, R, and S groups at 3 hours, 6 hours, 12 hours, and 18 hours revealed that amylase and proinflammatory and anti-inflammatory cytokine levels changed less in the R group than in the S group and less in the S group than in the SAP group. Based on the results of pairwise comparisons between the SAP, R, and S groups at four timepoints, the JAK2 mRNA and p-JAK2 protein expression levels in the pancreas and terminal ileum was less in the R group than in the SAP group, and the levels of STAT3 mRNA and p-STAT3 protein expression was greater in the SAP group than in the S group and greater in the S group than in the R group. Some studies have reported similar research conclusions [30, 31].

Zhu et al. [32] found that the JAK2/STAT3 pathway has an important role in the molecular mechanism of SAP-induced acute renal injury and that the suppression of the JAK2/STAT3 pathway could reduce TNF-*α* and IL-6 plasma levels. In a rat model of SAP-acute lung injury, Han et al. [33] demonstrated that dexamethasone treatment suppressed intercellular adhesion molecule-1 mRNA and protein expression via inhibiting IL-6 and TNF-*α*-induced JAK2/STAT3 activation.

The Chinese Materia Medica can ameliorate pancreatic and intestinal damage and inflammatory responses by inhibiting the JAK2/STAT3 signaling pathway. Our study suggested that DCQD acted as a JAK2 inhibitor and STAT3 inhibitor and markedly decreased the serum levels of TNF-*α*, IL-6, IL-4, and IL-10 and downregulated mRNA and protein levels of JAK2/STAT3 pathway components in the pancreas and terminal ileum. DCQD can restore gastrointestinal function by relieving enteroparalysis and facilitating bowel movement, inhibiting cytokine activity, resisting the inflammatory response, and relieving acute organ injury in SAP [9, 34]. Zhao et al. [35] reported that DCQD had good prospects in the treatment for SAP patient with acute respiratory distress syndrome in the clinic and was confirmed to inhibiting the production of inflammatory factors and mitigating lung injury in the SAP model.

## 5. Conclusion

The activation of JAK2/STAT3 signaling pathway may have a key influence on the pathogenesis of SAP-associated inflammatory cytokines and intestinal damage. DCQD could improve inflammatory cytokines and intestine injury in rats with SAP like JAK2 inhibitor and STAT3 inhibitor, which might be closely related to the inhibition of JAK2/STAT3 signaling pathway.

## Figures and Tables

**Table 1:** Comparisons of the dynamic serum amylase levels.

Group	3 hours	6 hours	12 hours	18 hours
SO	1589.5 ± 179.3	2910.5 ± 725.4^★^	1600.5 ± 252.7^★^	1637.1 ± 277.7
SAP	6329.7 ± 1496.3^♀^	9951.0 ± 1825.7^▲♂^	8523.9 ± 1359.9^▲☆^	6784.0 ± 795.6^▲◎^
R	3871.0 ± 1112.5^♀^	4689.4 ± 1059.3^■♂^	5390.6 ± 1009.1^■☆^	4754.9 ± 1197.8^■◎^
S	4114.5 ± 979.4^♀^	5579.6 ± 956.9^•♂^	6129.7 ± 1227.4^•☆^	5157.2 ± 1127.8^•◎^
DCQD	3219.5 ± 895.7^♀^	4272.8 ± 930.1^◢♂^	4917.5 ± 990.6^◢☆^	4037.8 ± 833.9^◢◎^

Note: The data are presented as U/L. ^★^In the SO group, 3 hours versus 6 hours and 6 hours versus 12 hours are significantly different (*P* < 0.05). ^▲^In the SAP group, 3 hours versus 6 hours, 6 hours versus 12 hours, and 12 hours versus 18 hours are significantly different (*P* < 0.05). ^■^In the R group, 3 hours versus 6 hours, 6 hours versus 12 hours, and 12 hours versus 18 hours are significantly different (*P* < 0.05). ^•^In the S group, 3 hours versus 6 hours, 6 hours versus 12 hours, and 12 hours versus 18 hours are significantly different (*P* < 0.05). ^◢^In the DCQD group, 3 hours versus 6 hours, 6 hours versus 12 hours, and 12 hours versus 18 hours are significantly different (*P* < 0.05). ^♀^At 3 hours, the SO group versus the SAP group, the SAP group versus the R group, the R group versus the S group, and the S group versus the DCQD group are significantly different. ^♂^At 6 hours, the SO group versus the SAP group, the SAP group versus the R group, the R group versus the S group, and the S group versus the DCQD group are significantly different. ^☆^At 12 hours, the SO group versus the SAP group, the SAP group versus the R group, the R group versus the S group, and the S group versus the DCQD group are significantly different. ^◎^At 18 hours, the SO group versus the SAP group, the SAP group versus the R group, the R group versus the S group, and the S group versus the DCQD group are significantly different. DCQD, Da-Cheng-Qi decoction; GAPDH, glyceraldehyde-3-phosphate dehydrogenase; IL-4, interleukin 4; IL-6, interleukin 6; IL-10 interleukin 10; JAK2, Janus kinase 2; p-JAK2, phosphorylated Janus kinase 2; p-STAT3, phosphorylated signal transducers and transcription 3; R, ruxolitinib-treated rats with severe acute pancreatitis; S, Stattic-treated rats with severe acute pancreatitis; SAP, severe acute pancreatitis; SO, sham operation; STAT3, signal transducers and transcription 3; TNF-*α*, tumor necrosis factor alpha.

**Table 2:** Comparisons of the dynamic TNF-*α* levels.

Group	3 hours	6 hours	12 hours	18 hours
SO	12.45 ± 3.32	18.01 ± 4.97^★^	15.29 ± 5.11^★^	13.93 ± 6.87
SAP	89.87 ± 16.37^♀^	148.56 ± 17.97^▲♂^	187.58 ± 20.09^▲☆^	163.22 ± 25.16^▲◎^
R	63.70 ± 19.65^♀^	98.94 ± 20.97^■♂^	147.25 ± 27.85^■☆^	124.85 ± 24.93^■◎^
S	71.21 ± 20.17^♀^	119.77 ± 23.39^•♂^	156.25 ± 23.17^•☆^	135.78 ± 26.67^•◎^
DCQD	53.85 ± 18.32^♀^	89.38 ± 20.47^◢♂^	131.87 ± 22.41^◢☆^	110.54 ± 25.01^◢◎^

Note: The data are presented as pg/mL. ^★^In the SO group, 3 hours versus 6 hours and 6 hours versus 12 hours are significantly different (*P* < 0.05). ^▲^In the SAP group, 3 hours versus 6 hours, 6 hours versus 12 hours, and 12 hours versus 18 hours are significantly different (*P* < 0.05). ^■^In the R group, 3 hours versus 6 hours, 6 hours versus 12 hours, and 12 hours versus 18 hours are significantly different (*P* < 0.05). ^•^In the S group, 3 hours versus 6 hours, 6 hours versus 12 hours, and 12 hours versus 18 hours are significantly different (*P* < 0.05).^◢^In the DCQD group, 3 hours versus 6 hours, 6 hours versus 12 hours, and 12 hours versus 18 hours are significantly different (*P* < 0.05). ^♀^ At 3 hours, the SO group versus the SAP group, the SAP group versus the R group, the R group versus the S group, and the S group versus the DCQD group are significantly different. ^♂^At 6 hours, the SO group versus the SAP group, the SAP group versus the R group, the R group versus the S group, and the S group versus the DCQD group are significantly different. ^☆^ At 12 hours, the SO group versus the SAP group, the SAP group versus the R group, the R group versus the S group, and the S group versus the DCQD group are significantly different. ^◎^ At 18 hours, the SO group versus the SAP group, the SAP group versus the R group, the R group versus the S group, and the S group versus the DCQD group are significantly different. DCQD, Da-Cheng-Qi decoction; GAPDH, glyceraldehyde-3-phosphate dehydrogenase; IL-4, interleukin 4; IL-6, interleukin 6; IL-10 interleukin 10; JAK2, Janus kinase 2; p-JAK2, phosphorylated Janus kinase 2; p-STAT3, phosphorylated signal transducers and transcription 3; R, ruxolitinib-treated rats with severe acute pancreatitis; S, Stattic-treated rats with severe acute pancreatitis; SAP, severe acute pancreatitis; SO, sham operation; STAT3, signal transducers and transcription 3; TNF-*α*, tumor necrosis factor alpha.

**Table 3:** Comparisons of the dynamic IL-6 levels.

Group	3 hours	6 hours	12 hours	18 hours
SO	25.45 ± 6.13	32.05 ± 7.64^★^	29.09 ± 7.87^★^	28.91 ± 8.79
SAP	89.87 ± 16.37^♀^	151.08 ± 15.37^▲♂^	213.29 ± 19.59^▲☆^	184.70 ± 15.86^▲◎^
R	57.06 ± 22.85^♀^	128.34 ± 19.93^■♂^	180.66 ± 29.31^■☆^	164.79 ± 29.48^■◎^
S	64.53 ± 19.74^♀^	139.86 ± 21.69^•♂^	199.57 ± 27.04^•☆^	177.02 ± 27.83^•◎^
DCQD	50.59 ± 22.57^♀^	120.82 ± 20.15^◢♂^	168.15 ± 24.86^◢☆^	141.30 ± 23.80^◢◎^

Note: The data are presented as pg/mL. ^★^In the SO group, 3 hours versus 6 hours and 6 hours versus 12 hours are significantly different (*P* < 0.05). ^▲^In the SAP group, 3 hours versus 6 hours, 6 hours versus 12 hours, and 12 hours versus 18 hours are significantly different (*P* < 0.05). ^■^In the R group, 3 hours versus 6 hours, 6 hours versus 12 hours, and 12 hours versus 18 hours are significantly different (*P* < 0.05). ^•^In the S group, 3 hours versus 6 hours, 6 hours versus 12 hours, and 12 hours versus 18 hours are significantly different (*P* < 0.05).^◢^In the DCQD group, 3 hours versus 6 hours, 6 hours versus 12 hours, and 12 hours versus 18 hours are significantly different (*P* < 0.05). ^♀^At 3 hours, the SO group versus the SAP group, the SAP group versus the R group, the R group versus the S group, and the S group versus the DCQD group are significantly different. ^♂^At 6 hours, the SO group versus the SAP group, the SAP group versus the R group, the R group versus the S group, and the S group versus the DCQD group are significantly different. ^☆^At 12 hours, the SO group versus the SAP group, the SAP group versus the R group, the R group versus the S group, and the S group versus the DCQD group are significantly different. ^◎^At 18 hours, the SO group versus the SAP group, the SAP group versus the R group, the R group versus the S group, and the S group versus the DCQD group are significantly different. DCQD, Da-Cheng-Qi decoction; GAPDH, glyceraldehyde-3-phosphate dehydrogenase; IL-4, interleukin 4; IL-6, interleukin 6; IL-10 interleukin 10; JAK2, Janus kinase 2; p-JAK2, phosphorylated Janus kinase 2; p-STAT3, phosphorylated signal transducers and transcription 3; R, ruxolitinib-treated rats with severe acute pancreatitis; S, Stattic-treated rats with severe acute pancreatitis; SAP, severe acute pancreatitis; SO, sham operation; STAT3, signal transducers and transcription 3; TNF-*α*, tumor necrosis factor alpha.

**Table 4:** Comparisons of the dynamic IL-4 levels.

Group	3 hours	6 hours	12 hours	18 hours
SO	12.99 ± 2.61	16.97 ± 4.37^★^	13.58 ± 3.93^★^	12.49 ± 3.21
SAP	31.29 ± 8.59^♀^	45.08 ± 15.37^▲♂^	58.85 ± 9.34^▲☆^	50.65 ± 11.06^▲◎^
R	23.79 ± 11.35^♀^	34.09 ± 14.03^■♂^	45.89 ± 16.95^■☆^	38.47 ± 13.06^■◎^
S	28.09 ± 12.17^♀^	40.65 ± 13.85^•♂^	50.19 ± 15.87^•☆^	41.02 ± 13.29^•◎^
DCQD	20.73 ± 9.20^♀^	31.37 ± 12.71^◢♂^	39.87 ± 15.00^◢☆^	31.85 ± 12. 71^◢◎^

Note: The data are presented as pg/mL. ^★^In the SO group, 3 hours versus 6 hours and 6 hours versus 12 hours are significantly different (*P* < 0.05). ^▲^In the SAP group, 3 hours versus 6 hours, 6 hours versus 12 hours, and 12 hours versus 18 hours are significantly different (*P* < 0.05). ^■^In the R group, 3 hours versus 6 hours, 6 hours versus 12 hours, and 12 hours versus 18 hours are significantly different (*P* < 0.05). ^•^In the S group, 3 hours versus 6 hours, 6 hours versus 12 hours, and 12 hours versus 18 hours are significantly different (*P* < 0.05).^◢^In the DCQD group, 3 hours versus 6 hours, 6 hours versus 12 hours, and 12 hours versus 18 hours are significantly different (*P* < 0.05). ^♀^At 3 hours, the SO group versus the SAP group, the SAP group versus the R group, the R group versus the S group, and the S group versus the DCQD group are significantly different. ^♂^At 6 hours, the SO group versus the SAP group, the SAP group versus the R group, the R group versus the S group, and the S group versus the DCQD group are significantly different. ^☆^At 12 hours, the SO group versus the SAP group, the SAP group versus the R group, the R group versus the S group, and the S group versus the DCQD group are significantly different. ^◎^At 18 hours, the SO group versus the SAP group, the SAP group versus the R group, the R group versus the S group, and the S group versus the DCQD group are significantly different. DCQD, Da-Cheng-Qi decoction; GAPDH, glyceraldehyde-3-phosphate dehydrogenase; IL-4, interleukin 4; IL-6, interleukin 6; IL-10 interleukin 10; JAK2, Janus kinase 2; p-JAK2, phosphorylated Janus kinase 2; p-STAT3, phosphorylated signal transducers and transcription 3; R, ruxolitinib-treated rats with severe acute pancreatitis; S, Stattic-treated rats with severe acute pancreatitis; SAP, severe acute pancreatitis; SO, sham operation; STAT3, signal transducers and transcription 3; TNF-*α*, tumor necrosis factor alpha.

**Table 5:** Comparisons of the dynamic IL-10 levels.

Group	3 hours	6 hours	12 hours	18 hours
SO	10.18 ± 3.08	15.93 ± 4.27^★^	12.07 ± 4.01^★^	11.29 ± 3.77
SAP	28.31 ± 9.91^♀^	41.01 ± 12.08^▲♂^	50.11 ± 11.89^▲☆^	44.47 ± 11.56^▲◎^
R	21.00 ± 11.28^♀^	35.57 ± 13.13^■♂^	46.19 ± 14.35^■☆^	38.47 ± 12.16^■◎^
S	18.14 ± 10.19^♀^	32.24 ± 12.57^•♂^	40.11 ± 12.34^•☆^	34.52 ± 11.25^•◎^
DCQD	15.80 ± 7.61^♀^	25.57 ± 12.01^◢♂^	39.07 ± 14.01^◢☆^	31.85 ± 12. 11^◢◎^

Note: The data are presented as pg/mL. ^★^In the SO group, 3 hours versus 6 hours and 6 hours versus 12 hours are significantly different (*P* < 0.05). ^▲^In the SAP group, 3 hours versus 6 hours, 6 hours versus 12 hours, and 12 hours versus 18 hours are significantly different (*P* < 0.05). ^■^In the R group, 3 hours versus 6 hours, 6 hours versus 12 hours, and 12 hours versus 18 hours are significantly different (*P* < 0.05). ^•^In the S group, 3 hours versus 6 hours, 6 hours versus 12 hours, and 12 hours versus 18 hours are significantly different (*P* < 0.05).^◢^In the DCQD group, 3 hours versus 6 hours, 6 hours versus 12 hours, and 12 hours versus 18 hours are significantly different (*P* < 0.05). ^♀^At 3 hours, the SO group versus the SAP group, the SAP group versus the R group, the R group versus the S group, and the S group versus the DCQD group are significantly different. ^♂^At 6 hours, the SO group versus the SAP group, the SAP group versus the R group, the R group versus the S group, and the S group versus the DCQD group are significantly different. ^☆^At 12 hours, the SO group versus the SAP group, the SAP group versus the R group, the R group versus the S group, and the S group versus the DCQD group are significantly different. ^◎^At 18 hours, the SO group versus the SAP group, the SAP group versus the R group, the R group versus the S group, and the S group versus the DCQD group are significantly different. DCQD, Da-Cheng-Qi decoction; GAPDH, glyceraldehyde-3-phosphate dehydrogenase; IL-4, interleukin 4; IL-6, interleukin 6; IL-10 interleukin 10; JAK2, Janus kinase 2; p-JAK2, phosphorylated Janus kinase 2; p-STAT3, phosphorylated signal transducers and transcription 3; R, ruxolitinib-treated rats with severe acute pancreatitis; S, Stattic-treated rats with severe acute pancreatitis; SAP, severe acute pancreatitis; SO, sham operation; STAT3, signal transducers and transcription 3; TNF-*α*, tumor necrosis factor alpha.

**Table 6:** Comparisons of the protein expression of p-JAK2 in the pancreas.

Group	3 hours	6 hours	12 hours	18 hours
SO	0.412 ± 0.075	0.487 ± 0.112^★^	0.556 ± 0.122^★^	0.454 ± 0.107^★^
SAP	0.733 ± 0.186^♀a^	0.980 ± 0.199^▲♂a^	1.223 ± 0.223^▲☆a^	0.935 ± 0.197^▲◎a^
R	0.622 ± 0.112^♀^	0.881 ± 0.181^■♂^	1.110 ± 0.207^■☆^	0.855 ± 0.184^■◎^
S	0.715 ± 0.190^♀a^	0.980 ± 0.192^•♂a^	1.231 ± 0.214^•☆a^	0.941 ± 0.189^•◎a^
DCQD	0.683 ± 0.114^♀^	0.871 ± 0.187^◢♂^	1.101 ± 0.211^◢☆^	0.871 ± 0.193^◢◎^

Note: The data are presented as the p-JAK2/GAPDH ratio. ^★^In the SO group, 3 hours versus 6 hours, 6 hours versus 12 hours, and 12 hours versus 18 hours are significantly different (*P* < 0.05). ^▲^In the SAP group, 3 hours versus 6 hours, 6 hours versus 12 hours, and 12 hours versus 18 hours are significantly different (*P* < 0.05). ^■^In the R group, 3 hours versus 6 hours, 6 hours versus 12 hours, and 12 hours versus 18 hours are significantly different (*P* < 0.05). ^•^In the S group, 3 hours versus 6 hours, 6 hours versus 12 hours, and 12 hours versus 18 hours are significantly different (*P* < 0.05).^◢^In the DCQD group, 3 hours versus 6 hours, 6 hours versus 12 hours, and 12 hours versus 18 hours are significantly different (*P* < 0.05). ^♀^At 3 hours, the SO group versus the SAP group, the SAP group versus the R group, the R group versus the S group, and the S group versus the DCQD group are significantly different. ^♂^At 6 hours, the SO group versus the SAP group, the SAP group versus the R group, the R group versus the S group, and the S group versus the DCQD group are significantly different. ^☆^At 12 hours, the SO group versus the SAP group, the SAP group versus the R group, the R group versus the S group, and the S group versus the DCQD group are significantly different. ^◎^At 18 hours, the SO group versus the SAP group, the SAP group versus the R group, the R group versus the S group, and the S group versus the DCQD group are significantly different. ^a^At the same timepoints, the SAP group versus the S group was not significantly different (*P* > 0.05). DCQD, Da-Cheng-Qi decoction; GAPDH, glyceraldehyde-3-phosphate dehydrogenase; IL-4, interleukin 4; IL-6, interleukin 6; IL-10 interleukin 10; JAK2, Janus kinase 2; p-JAK2, phosphorylated Janus kinase 2; p-STAT3, phosphorylated signal transducers and transcription 3; R, ruxolitinib-treated rats with severe acute pancreatitis; S, Stattic-treated rats with severe acute pancreatitis; SAP, severe acute pancreatitis; SO, sham operation; STAT3, signal transducers and transcription 3; TNF-*α*, tumor necrosis factor alpha.

**Table 7:** Comparisons of the protein expression of p-JAK2 in the terminal ileum.

Group	3 hours	6 hours	12 hours	18 hours
SO	0.446 ± 0.077	0.501 ± 0.085^★^	0.574 ± 0.085^★^	0.481 ± 0.095^★^
SAP	0.802 ± 0.178^♀a^	0.991 ± 0.182^▲♂a^	1.345 ± 0.246^▲☆a^	0.921 ± 0.181^▲◎a^
R	0.661 ± 0.121^♀^	0.910 ± 0.133^■♂^	1.174 ± 0.221^■☆^	0.885 ± 0.204^■◎^
S	0.775 ± 0.168^♀a^	0.950 ± 0.142^•♂a^	1.321 ± 0.233^•☆a^	0.922 ± 0.185^•◎a^
DCQD	0.671 ± 0.127^♀^	0.935 ± 0.142^◢♂^	1.211 ± 0.212^◢☆^	0.851 ± 0.196^◢◎^

Note: The data are presented as the p-JAK2/GAPDH ratio. ^★^In the SO group, 3 hours versus 6 hours, 6 hours versus 12 hours, and 12 hours versus 18 hours are significantly different (*P* < 0.05).^▲^In the SAP group, 3 hours versus 6 hours, 6 hours versus 12 hours, and 12 hours versus 18 hours are significantly different (*P* < 0.05). ^■^In the R group, 3 hours versus 6 hours, 6 hours versus 12 hours, and 12 hours versus 18 hours are significantly different (*P* < 0.05).^•^In the S group, 3 hours versus 6 hours, 6 hours versus 12 hours, and 12 hours versus 18 hours are significantly different (*P* < 0.05).^◢^In the DCQD group, 3 hours versus 6 hours, 6 hours versus 12 hours, and 12 hours versus 18 hours are significantly different (*P* < 0.05). ^♀^At 3 hours, the SO group versus the SAP group, the SAP group versus the R group, the R group versus the S group, and the S group versus the DCQD group are significantly different. ^♂^At 6 hours, the SO group versus the SAP group, the SAP group versus the R group, the R group versus the S group, and the S group versus the DCQD group are significantly different. ^☆^At 12 hours, the SO group versus the SAP group, the SAP group versus the R group, the R group versus the S group, and the S group versus the DCQD group are significantly different. ^◎^At 18 hours, the SO group versus the SAP group, the SAP group versus the R group, the R group versus the S group, and the S group versus the DCQD group are significantly different. ^a^At the same timepoints, the SAP group versus the S group are not significantly different (*P* > 0.05). DCQD, Da-Cheng-Qi decoction; GAPDH, glyceraldehyde-3-phosphate dehydrogenase; IL-4, interleukin 4; IL-6, interleukin 6; IL-10 interleukin 10; JAK2, Janus kinase 2; p-JAK2, phosphorylated Janus kinase 2; p-STAT3, phosphorylated signal transducers and transcription 3; R, ruxolitinib-treated rats with severe acute pancreatitis; S, Stattic-treated rats with severe acute pancreatitis; SAP, severe acute pancreatitis; SO, sham operation; STAT3, signal transducers and transcription 3; TNF-*α*, tumor necrosis factor alpha.

**Table 8:** Comparisons of the protein expression of p-STAT3 in the pancreas.

Group	3 hours	6 hours	12 hours	18 hours
SO	0.302 ± 0.055	0.383 ± 0.060^★^	0.412 ± 0.065^★^	0.343 ± 0.056^★^
SAP	0.621 ± 0.166^♀^	0.877 ± 0.169^▲♂^	1.131 ± 0.222^▲☆^	0.871 ± 0.170^▲◎^
R	0.570 ± 0.129^♀^	0.734 ± 0.138^■♂^	0.990 ± 0.191^■☆^	0.741 ± 0.190^■◎^
S	0.492 ± 0.107^♀^	0.605 ± 0.124^•♂^	0.851 ± 0.180^•☆^	0.632 ± 0.127^•◎^
DCQD	0.433 ± 0.094^♀^	0.582 ± 0.121^◢♂^	0.792 ± 0.196^◢☆^	0.618 ± 0.121^◢◎^

Note: The data are presented as the p-STAT3/GAPDH ratio. ^★^In the SO group, 3 hours versus 6 hours, 6 hours versus 12 hours, and 12 hours versus 18 hours are significantly different (*P* < 0.05). ^▲^In the SAP group, 3 hours versus 6 hours, 6 hours versus 12 hours, and 12 hours versus 18 hours are significantly different (*P* < 0.05). ^■^In the R group, 3 hours versus 6 hours, 6 hours versus 12 hours, and 12 hours versus 18 hours are significantly different (*P* < 0.05). ^•^In the S group, 3 hours versus 6 hours, 6 hours versus 12 hours, and 12 hours versus 18 hours are significantly different (*P* < 0.05).^◢^In the DCQD group, 3 hours versus 6 hours, 6 hours versus 12 hours, and 12 hours versus 18 hours are significantly different (*P* < 0.05). ^♀^At 3 hours, the SO group versus the SAP group, the SAP group versus the R group, the R group versus the S group, and the S group versus the DCQD group are significantly different. ^♂^At 6 hours, the SO group versus the SAP group, the SAP group versus the R group, the R group versus the S group, and the S group versus the DCQD group are significantly different. ^☆^At 12 hours, the SO group versus the SAP group, the SAP group versus the R group, the R group versus the S group, and the S group versus the DCQD group are significantly different. ^◎^At 18 hours, the SO group versus the SAP group, the SAP group versus the R group, the R group versus the S group, and the S group versus the DCQD group are significantly different. DCQD, Da-Cheng-Qi decoction; GAPDH, glyceraldehyde-3-phosphate dehydrogenase; IL-4, interleukin 4; IL-6, interleukin 6; IL-10 interleukin 10; JAK2, Janus kinase 2; p-JAK2, phosphorylated Janus kinase 2; p-STAT3, phosphorylated signal transducers and transcription 3; R, ruxolitinib-treated rats with severe acute pancreatitis; S, Stattic-treated rats with severe acute pancreatitis; SAP, severe acute pancreatitis; SO, sham operation; STAT3, signal transducers and transcription 3; TNF-*α*, tumor necrosis factor alpha.

**Table 9:** Comparison of the protein expression of p-STAT3 in the terminal ileum.

Group	3 hours	6 hours	12 hours	18 hours
SO	0.342 ± 0.065	0.403 ± 0.067^★^	0.443 ± 0.074^★^	0.364 ± 0.057^★^
SAP	0.691 ± 0.144^♀^	0.912 ± 0.175^▲♂^	1.291 ± 0.214^▲☆^	0.890 ± 0.168^▲◎^
R	0.645 ± 0.120^♀^	0.813 ± 0.124^■♂^	0.984 ± 0.187^■☆^	0.780 ± 0.170^■◎^
S	0.577 ± 0.110^♀^	0.710 ± 0.123^•♂^	0.861 ± 0.165^•☆^	0.700 ± 0.162^•◎^
DCQD	0.557 ± 0.107^♀^	0.673 ± 0.129^◢♂^	0.808 ± 0.175^◢☆^	0.679 ± 0.167^◢◎^

Note: The data are presented as the p-STAT3/GAPDH ratio. ^★^In the SO group, 3 hours versus 6 hours, 6 hours versus 12 hours, and 12 hours versus 18 hours are significantly different (*P* < 0.05). ^▲^In the SAP group, 3 hours versus 6 hours, 6 hours versus 12 hours, and 12 hours versus 18 hours are significantly different (*P* < 0.05). ^■^In the R group, 3 hours versus 6 hours, 6 hours versus 12 hours, and 12 hours versus 18 hours are significantly different (*P* < 0.05). ^•^In the S group, 3 hours versus 6 hours, 6 hours versus 12 hours, and 12 hours versus 18 hours are significantly different (*P* < 0.05). ^◢^In the DCQD group, 3 hours versus 6 hours, 6 hours versus 12 hours, and 12 hours versus 18 hours are significantly different (*P* < 0.05). ^♀^At 3 hours, the SO group versus the SAP group, the SAP group versus the R group, the R group versus the S group, and the S group versus the DCQD group are significantly different. ^♂^At 6 hours, the SO group versus the SAP group, the SAP group versus the R group, the R group versus the S group, and the S group versus the DCQD group are significantly different. ^☆^At 12 hours, the SO group versus the SAP group, the SAP group versus the R group, the R group versus the S group, and the S group versus the DCQD group are significantly different. ^◎^At 18 hours, the SO group versus the SAP group, the SAP group versus the R group, the R group versus the S group, and the S group versus the DCQD group are significantly different. DCQD, Da-Cheng-Qi decoction; GAPDH, glyceraldehyde-3-phosphate dehydrogenase; IL-4, interleukin 4; IL-6, interleukin 6; IL-10 interleukin 10; JAK2, Janus kinase 2; p-JAK2, phosphorylated Janus kinase 2; p-STAT3, phosphorylated signal transducers and transcription 3; R, ruxolitinib-treated rats with severe acute pancreatitis; S, Stattic-treated rats with severe acute pancreatitis; SAP, severe acute pancreatitis; SO, sham operation; STAT3, signal transducers and transcription 3; TNF-*α*, tumor necrosis factor alpha.

## Data Availability

The materials and data are available from the corresponding author upon request.
